# Combatting Neurophobia: A Proposed Preliminary Educational Model to Promote Neurophilia

**DOI:** 10.7759/cureus.51855

**Published:** 2024-01-08

**Authors:** Richard I Suarez, Jenny Fortun

**Affiliations:** 1 Herbert Wertheim College of Medicine, Florida International University, Miami, USA

**Keywords:** pre-clinical medical education, neurology, neurology medical education, medical educaiton, neurophobia

## Abstract

When we reflect on medical education as a whole, novelty in structure and content promotes growth and enhances student outcomes. The teaching of neurology is no different and presents a more unique hurdle in its instruction considering the well-described phenomenon of *neurophobia*. With the burden of neurological diseases on the rise, there is a heightened demand for medical educators to understand the possible causes of this educational misalignment and implement solutions necessary to ensure adequate education of students. In this study, we describe a novel approach to neurology education for second-year medical students to stimulate *neurophilia*, incorporating evidence-based approaches within the identified areas-Active Learning Pedagogies, Diagnostic and Clinical Reasoning, Use of Technology, Field Exposure and Mentorship, and Innovation. Students demonstrated superior academic performance on the National Board of Medical Examiners (NBME) neurology assessments and generally positive feedback on the use of innovative approaches to teaching and learning. Overall, we propose this method of teaching neurology as a model educational platform that aims to reduce *neurophobia* and promote *neurophilia*.

## Introduction

Current medical education trends emphasize the need to teach basic sciences in a way that fosters its application into clinical practice, ensuring students adequately comprehend and relate the medical knowledge necessary to be clinicians. The field of neurology is not an exception. *Neurophobia* is defined as “a fear of the neural sciences and clinical neurology that is due to the student’s inability to apply their knowledge of basic sciences to clinical situations” [1]. With the global burden of neurological disease continuing to trend upward [2], elucidating the reasons for *neurophobia* and strategies for the promotion of its counterpart - *neurophilia *- are necessary to better teach students about neurology.

Different studies have examined the various reasons for neurophobia, which we have grouped into four major categories: complexity, emotional toll, clinical experience, and education. *Complexity* refers to reports of students finding the subject material challenging, particularly neuroanatomy, as well as the density of the physical exam and diagnostic evaluation [3-7]. *Emotional toll* encompasses the overall sentiment of increased suffering of patients with neurological disorders, which weighs heavily on providers, alongside the perception of many of these conditions being uncurable at this time [3-7]. The studies also present the fact that students did not have enough exposure to neurology to understand how it functions in clinical practice (*clinical experience*), including interacting with patients and limited engagement from community practitioners [3-7]. Finally, insufficient or inadequate instruction has been found to contribute to the disengagement and lack of interest in neurology, which could impact negatively the clinical competence and proficiency of future clinicians [3-7].

Various interventions have been reported to enhance neurology education, which we have grouped into five categories based on similarity: Active Learning Pedagogies, Diagnostic and Clinical Reasoning, Use of Technology, Field Exposure and Mentorship, and Innovation. For example, team-based and case-based learning (CBL) increased confidence and improved student performance on exams [8-10]. Implementation of diagnostic and clinical reasoning into neurology curriculum, such as hypothesis-driven assessment, alongside screening examinations, increased the likelihood of properly identifying abnormal findings [11]. The use of multimedia educational modules for neuroanatomy and neuroscience provided flexibility and adaptability to the teaching of medical students, expanding how the material could be disseminated, and resulting in similar (or enhanced) outcomes for students [12]. Furthermore, by increasing interactions with providers and patients, students were more likely to enjoy the material and perform better on clinical and summative examinations [13-14]. Finally, innovative approaches, such as the development of a neuroanatomy elective and/or clinical reasoning practice sessions for the application of the basic science principles, expanded on the traditional teaching pedagogies, inviting more learners [15-16].

The current education literature highlights the practices described above in isolation and does not provide an example curriculum that integrates all the major recommended interventions. Moreover, articles that make curricular recommendations are about clerkship education of medical students and/or provide broad suggestions for the construction of these courses [13,17]. Here, we propose a novel, preliminary neurology education model used for second-year medical students (preclinical) that follows evidence-based approaches, combining elements of the five interventions described earlier.

## Technical report

Proposed educational model

**Figure 1:**
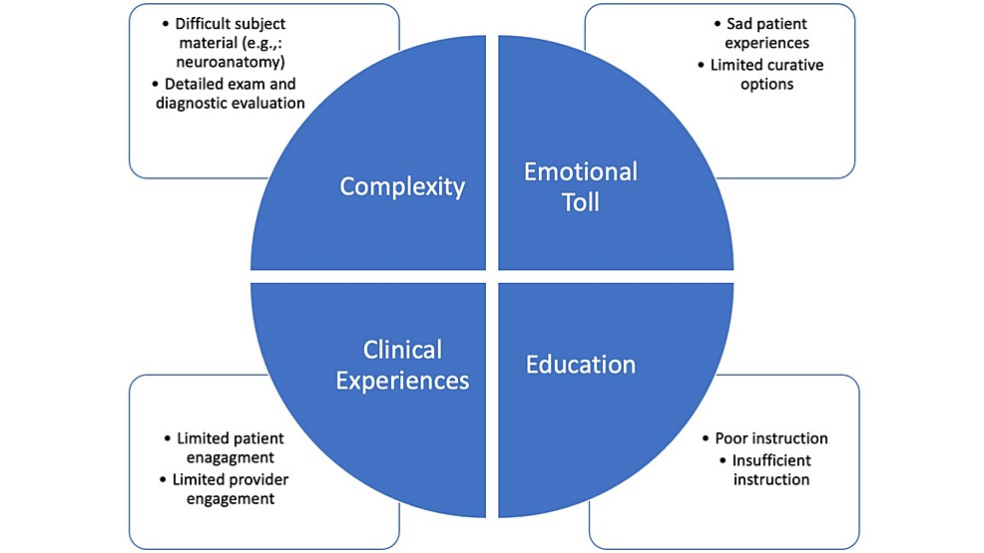
Tenets of neurophobia. The figure is author-generated. Information adapted from various sources [3-7].

**Figure 2:**
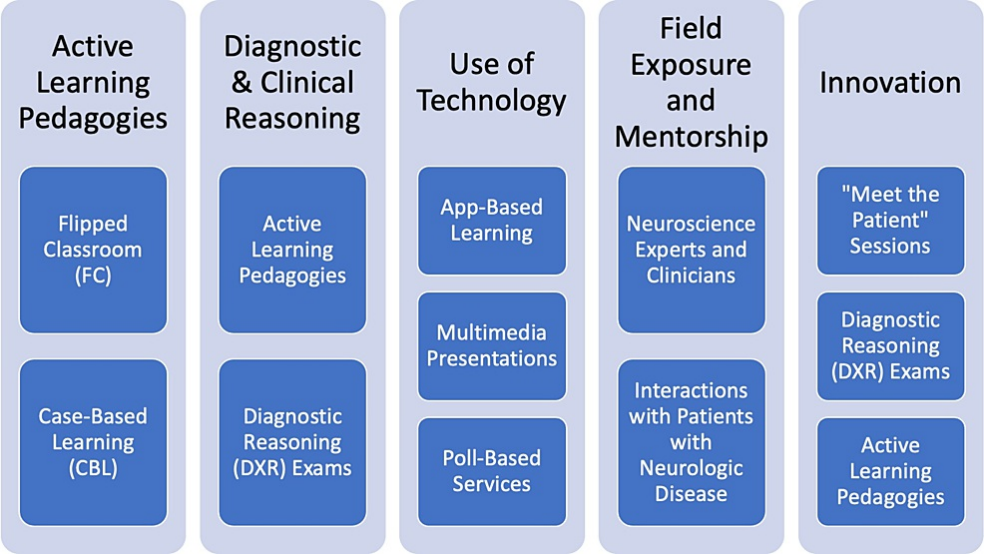
Proposed educational model alignment with evidence-based recommendations. The figure is author-generated. Information adapted from various sources [8-16]. FC, flipped classroom; CBL, case-based learning; DXR, diagnostic reasoning exam

The main propagators responsible for *neurophobia* are seen throughout neurology medical education, emphasizing the need to develop new ways to educate students (Figure 1). In this model, the five identified intervention strategies - active learning pedagogies, diagnostic and clinical reasoning, use of technology, field exposure and mentorship, and innovation - are well-represented. Figure 2 summarizes the methods used within this curriculum that satisfy these evidence-based recommendations.

Active Learning Pedagogies

These teaching methodologies ensure that students are active participants in their education rather than passive recipients. Although several active learning strategies exist, our proposed curriculum uses flipped classrooms (FC) and CBL.

During FC sessions, students are provided with materials to review independently before a session. They then present to the class, prepared to apply this information through interactive questions and case-based problems. In this course, students were provided with a study guide and voice-over presentation to prepare for the session and were expected to complete a graded readiness quiz before the session to support their preparation. FCs were used to cover the following topics: neurophysiology (action potentials, synaptic transmission, and neuronal communication), anesthetic agents (anxiolytics and hypnotics, nicotinic receptor modulators, and inhaled and local anesthetics), antibiotics, blood supply, and four different FCs aimed at localizing lesions (spinal cord, motor pathways, brainstem, vision, and eye movements). 

CBLs also allow students to apply information but following one single case. Here, students are expected to go through patient scenarios and subsequently ask questions as each case progresses. This encourages students to think about the patient factors, alongside the basic science concepts, and fosters understanding of the connection between the two as they relate to patient care. In this course, there were five CBLs. Each CBL either dealt with the identification of the cause of neurological presentation and the brain area damaged for a multitude of pathologies (strokes, seizures, tumors, etc.) or a theoretical patient scenario where they applied principles learned up to that point to develop and refine a differential diagnosis, interpret a diagnostic test, and explain the pathophysiology of disease and management.

The rationale behind the inclusion of these topics into an FC or a CBL was to emphasize understanding through application and enhance retention through preparation and demonstration of knowledge. This is particularly true when concerned with material that tends to be more difficult for students, including localizing the lesions, strokes (especially in the brainstem), and vision and eye movement disorders.

Diagnostic and Clinical Reasoning

Clinicians are expected to apply their understanding of clinical presentation, pathophysiology, and diagnostic evaluation to patient care. In addition to creating opportunities to develop and practice these skills, such as the CBLs and FCs described, it is critical to incorporate adequate assignments to assess this.

In this course, we included a summative written final exam known as a Diagnostic Reasoning (DXR) exam [18]. The DXR exam consists of a clinical case presentation that follows progressive disclosure, with a format similar to the CBLs. Each part contains an open-ended question (OEQ) designed to test critical thinking and diagnostic-reasoning skills and has backward navigation disabled. Students are expected to develop a broad differential diagnosis, refine it as they obtain more information, propose, and interpret diagnostic tests, and explain key elements of pathophysiology and patient management. Each part is graded independently: if students did not know the answer to the prior section or may have been going down the incorrect path, they are not penalized in every subsequent part. Overall, the intent is for students to reason through a patient case from initial exposure to diagnosis and management as they will need to do upon graduation.

Use of Technology

With the advent of the Internet, web-based learning platforms, simulations, and online tools, students can expand their horizons when it concerns what and how they learn. Thus, it is important to be able to integrate these processes within education to encourage engaged, enthusiastic, and forward-thinking learning. In this curriculum, students were presented with app-based learning tools, online recordings for independent studies, and poll-based services.

The app called *Complete Anatomy* is a service that allows students to visualize anatomical structures in 3D [19]. Here, students can look at organ systems and specific features of different systems together (to understand the many interactions) or in isolation, such as studying only the brainstem rather than the spinal cord, cerebrum, and cerebellum. Students are also able to project these structures using virtual reality (VR) to help with real-world visualization. The app offers multiple mechanisms of interaction. This provides another means of learning anatomy without necessarily having to be in a laboratory. This was used adjuvant to cadavers, other specimens, and plastic models.

Students throughout the course were provided with online recordings of lectures that they were responsible for viewing on their own time for independent study. These included the following lectures: review of neuroanatomy, neurologic diagnostic tests, histology of the nervous system, hypothalamus and thalamus, and rare neurologic tumors. In addition, all in-person lectures were recorded. Multimedia presentations available online to students offer flexibility in terms of the viewing and review of lectures, as well as allowing for more in-class presentations of more high-yield concepts, including stroke and lesion localizations.

Throughout the various types of sessions, including lectures, students were engaged using poll questions. Here, students would log in to an online server connected to the presentation where they could directly interact with the professor. The class performance and percentiles of each answer choice were displayed, providing an opportunity to explore student understanding and clarify confusion. Moreover, key neurological signs and testing were shown in class using a variety of videos, in addition to demonstrations.

Field Exposure and Mentorship

Being able to be taught by experts in the field, including neurologists, neurosurgeons, and patients themselves, serves to demonstrate practical application to the clinical arena. With this in mind, this course accomplished this through the lecturers and facilitators of the various sessions, as well as interactions with real patients.

Almost every lecture or session was led by a neurologist, neuroscientist, neurosurgeon, or other clinician related to the topic at hand. Furthermore, students had the opportunity to interact with patients with neurologic disorders in *Meet the Patient* sessions [20]. Here, patients shared their stories, and their symptoms provided students with the chance to not only gather patient perspectives on living with these conditions but also learn about the causes and relate them to their presentation. Students have the opportunity to ask questions to the patients as their story unfolds and are expected to answer questions about the patient's presentation. In this way, students are not solely learning these neurological concepts in isolation, but rather also having exposure to the humanistic aspects of medicine, serving as a reminder of the reasons they learn this information in the first place.

Innovation

The ever-changing atmosphere of medicine lends itself to being a field always in need of creativity to encourage appropriate knowledge acquisition and adequate application to clinical care. In this course, innovation can be seen through the *Meet the Patient* sessions, targeted FC and CBL sessions, and DXR exams. The incorporation of a Jeopardy session also serves as an example of unique methods of instruction and engagement that were used to encourage learning and application of material.

Overall, neurology education demands the use of evidence-based strategies to disrupt the occurrence of *neurophobia*. We have proposed an educational model that incorporates the five key areas that have been shown to help alleviate this phenomenon as a possible mechanism that can be replicated at other institutions.

Student reception and performance

It is important to consider proposed curricular changes in conjunction with student perception and impact on performance. Our preliminary results indicate a positive student reception and possible knowledge gains. We compared the class of 2024 (current model) with the class of 2023. Compared to the prior class, the current model has more active learning (four additional FCs and three additional CBLs), a *Meet the Patient* session and more time dedicated to neuroanatomy (in lectures, laboratories, and using Complete Anatomy). Additionally, the rest of the FCs and CBLs that were used before were modified based on student feedback. On the Likert Scale, where 1 signified strongly disagree and 5 denoted strongly agree, each category averaged approximately 4.31 (compared to 3.89 in the prior iteration of the course). The majority of the students felt that the course content matched the learning objectives, that the teaching methods used throughout the course fostered their learning, and that the assignments or activities were relevant and supported their learning. Another strength noted by the students was the course director’s ability to communicate and direct the course. In terms of the remarks from the students, most of the critical comments were related to the organization of the course, as well as the length of the exams. However, many commented on the challenging nature of the material, citing this as the problem they faced during the course rather than the different instructional elements used throughout.

Table 1 shows the performance of the two groups on their midterm, final, and DXR exams. Both their midterm and final exams were derived from the National Board of Medical Examiners (NBME) question bank, which consists of retired questions from previous versions of the licensing exams students are expected to pass before graduation (i.e., the United States Medical Licensing Examination [USMLE] Step 1). When compared to the national averages of students who were assessed with the same set of questions, both classes demonstrated above-average performance on both the midterm and final NBME exams. The DXR exam consists of about eight to 10 questions surrounding a specific case and requires students to answer questions related to differential diagnosis, diagnostic tests, pathophysiology, and management. Overall, the two groups performed well, with the average for the exam being 84% and 86% for the classes of 2024 and 2023, respectively. Of note, although all exams were different between the cohorts, the levels of complexity were similar.

**Table 1:** Class of 2023 and class of 2024 examination performance. n, number of questions per assessment; DXR, Diagnostic Reasoning; NBME, National Board of Medical Examiners

Exam	Class of 2023 performance (%)	Class of 2024 performance (%)
Midterm NBME	86 ± 7 (*n *= 53)	84 ± 7 (*n *= 80)
National average	77	76
Final NBME	85 ± 7 (*n *= 130)	84 ± 5 (*n *= 120)
National average	75	76
DXR	86 ± 7 (*n *= 8)	84 ± 8 (*n *= 10)

## Discussion

Extensive research has demonstrated how a general dislike for neurology is divided into four different categories: complexity, emotional toll, clinical experience, and education [3-7]. Different areas were identified that could be used to combat this phenomenon, including active learning pedagogies, diagnostic and clinical reasoning, use of technology, field exposure and mentorship, and innovation [8-9,11-12,14-16]. Though there are different ways to approach this issue, our preliminary educational model incorporates evidence-based recommendations and can serve as an example for programs to see how these different elements interact and overlap with one another. The decisions behind the fundamentals of our program were rooted in the complexity of the topic, the perception of the students, and the malleability of the concepts to fit into more unique methods of teaching, such as the active learning pedagogies, Meet the Patient sessions, or Jeopardy, among others. For this reason, we sought inspiration from student feedback, faculty expertise, and current practices in the medical education sphere to develop this program.

Our preliminary results showed increased student satisfaction with the new curriculum. Students found the course challenging, yet felt that the various aspects of the curriculum adhered to the learning objectives and the content of the assessments. The mean performance in all exams was high and above the national average for NBME exams. Although the mean in NBME exams was slightly higher before the intervention, this difference was not statistically different. In addition, all NBME and DXR exams were different among cohorts, and thus, a comparison cannot be made. Even though our preliminary results are encouraging, they are not enough to conclude that the intervention resulted in significant knowledge gains or the identified change in attitudes compared to the old curriculum. Further research is needed in this area to validate these claims.

As with every proposed educational model, there are limitations. For one, it requires significant faculty and student involvement. Faculty members would need to be trained in the pedagogies needed to lead active learning sessions, including FCs, CBLs, and Meet the Patient sessions. Similarly, for the inclusion of DXR exams (or an equivalent), several experts are needed to score the exam, and they all need to receive extensive faculty development. This would also stress the importance of strong community and hospital connections to recruit neuroscience faculty, especially neurologists and neuroscientists. The students of the course would also have expectations to engage more so than they may be used to or previously expected to with the prior preparation needed for the various interactive sessions, as well as the progressive disclosure essay exams. Furthermore, this model demands technology, and not all medical schools may have the capabilities to incorporate these elements, such as the Complete Anatomy application or the recording and uploading of lectures for student viewing. This type of curriculum lends itself to collaboration among faculty and students to learn how best to teach and assess neurology to develop a program that works best at the institution. Additionally, this study is limited as it compares two classes before and after incorporation and specifically looks at second-year medical students.

## Conclusions

Overall, we proposed a model to ameliorate *neurophobia* through the incorporation of the five evidence-based recommendations. Our preliminary results suggest its benefit expressed by students in their outlook and performance outcomes. We believe our format can be transferable to the teaching of neurology in other schools, as well as other organ system disciplines. We encourage other instructors at different medical schools to consider incorporating a program such as this that would aim to promote *neurophilia* among their students. This program can serve as an example of how to include these elements into a curriculum and offers various areas of flexibility that encourage modification to fit the unique needs and limitations of the institution. Future research is needed to investigate the longitudinal impact of this curriculum, including its effects on performance in clerkships and national licensing exams. Additionally, exploring the potential integration of similar models at different levels of medical education, such as in the first or third-fourth years, would be valuable.
